# Inhibition of the galactosyltransferase C1GALT1 reduces osteosarcoma cell proliferation by interfering with ERK signaling and cell cycle progression

**DOI:** 10.1038/s41417-024-00773-9

**Published:** 2024-04-15

**Authors:** Kentaro Watanabe, Keiji Tasaka, Hideto Ogata, Shota Kato, Hiroo Ueno, Katsutsugu Umeda, Tomoya Isobe, Yasuo Kubota, Masahiro Sekiguchi, Shunsuke Kimura, Aiko Sato-Otsubo, Mitsuteru Hiwatari, Tetsuo Ushiku, Motohiro Kato, Akira Oka, Satoru Miyano, Seishi Ogawa, Junko Takita

**Affiliations:** 1 Department of Pediatrics, Graduate School of Medicine, The University of Tokyo, Tokyo, Japan; 2 Department of Pediatrics, Graduate School of Medicine, Kyoto University, Kyoto, Japan; 3 Department of Pathology and Tumor Biology, Graduate School of Medicine, Kyoto University, Kyoto, Japan; 4 Department of Pediatrics, Hiroshima University Graduate School of Biomedical Sciences, Hiroshima, Japan; 5 Department of Pediatrics, Teikyo University, School of Medicine, Tokyo, Japan; 6 Department of Pathology, Graduate School of Medicine, The University of Tokyo, Tokyo, Japan; 7 Department of Integrated Analytics, M&D Data Science Center, Tokyo Medical and Dental University, Tokyo, Japan; 8 Department of Pathology and Tumor Biology, Institute for the Advanced Study of Human Biology (WPI-ASHBi), Kyoto University, Kyoto, Japan

**Keywords:** Bone cancer, Gene expression, Cell biology, Cancer genetics

## Abstract

Novel therapeutic strategies are urgently required for osteosarcoma, given the early age at onset and persistently high mortality rate. Modern transcriptomics techniques can identify differentially expressed genes (DEGs) that may serve as biomarkers and therapeutic targets, so we screened for DEGs in osteosarcoma. We found that osteosarcoma cases could be divided into fair and poor survival groups based on gene expression profiles. Among the genes upregulated in the poor survival group, siRNA-mediated knockdown of the glycosylation-related gene *C1GALT1* suppressed osteosarcoma cell proliferation in culture. Gene expression, phosphorylation, and glycome array analyses also demonstrated that *C1GALT1* is required to maintain ERK signaling and cell cycle progression. Moreover, the C1GALT1 inhibitor itraconazole suppressed osteosarcoma cell proliferation in culture, while doxycycline-induced shRNA-mediated knockdown reduced xenograft osteosarcoma growth in mice. Elevated *C1GALT1* expression is a potential early predictor of poor prognosis, while pharmacological inhibition may be a feasible treatment strategy for osteosarcoma.

## Introduction

Osteosarcoma is most frequent in teenagers and the most common bone tumor across age groups [1]. The EURAMOS-1 study reported that the 5-year event-free survival (EFS) of patients with localized disease at initial diagnosis was 60%, while that of patients with metastases at initial diagnosis was only 28% [2]. Since introducing three-drug chemotherapy (methotrexate, adriamycin, and cisplatin) in the 1980s, various additional drugs have been investigated for osteosarcoma, but these have not significantly improved outcomes [3–5]. Therefore, it is essential to develop targeted therapies based on a deeper understanding of molecular pathogenesis.

To develop new targeted therapies for osteosarcoma and other carcinomas, numerous attempts have been made to identify driver mutations by genomic analysis. However, it has been reported that mutations in osteosarcoma are mainly in tumor suppressor genes [6], precluding the use of inhibitory drugs as targeted therapies. Efforts have also been made to elucidate the molecular pathogenesis of osteosarcoma using mRNA and miRNA analyses [7, 8], but these studies have yet to identify reliable biomarkers or direct therapeutic targets.

Most previous reports on gene expression profiling in osteosarcoma have compared groups stratified based on predefined clinical characteristics, such as the presence or absence of metastases at the initial diagnosis. However, this method is not optimal for identifying heretofore unknown biological properties contributing to differences in disease course, treatment response, or outcome. Since there is rarely a simple association between a given clinical characteristic and biological property, tumor samples with unique biological properties potentially predictive of prognosis or providing clues to novel treatment strategies will be grouped with samples defined only by similar clinical characteristics. Thus, the magnitudes of such biological differences will be attenuated in whole-group analysis. To circumvent this problem, it may be useful to first divide patients into groups according to biological properties, such as unique gene expression profiles, using unsupervised (unbiased) methods. In addition, the robustness and stability of clustering can be substantiated by combining conventional genomics techniques with consensus clustering, which evaluates cluster stability by iterating the clustering of randomly extracted subsets [9, 10].

Based on these considerations, we analyzed expression array datasets from osteosarcoma patients using unsupervised consensus clustering to identify new biological groupings and associated clinical characteristics. This strategy identified the galactosyltransferase Core 1 synthase glycoprotein-N-acetylgalactosamine 3-beta-galactosyltransferase 1 (C1GALT1) as a promising biomarker of poor prognosis and a feasible treatment target for osteosarcoma.

## Materials and Methods

### Obtaining public datasets

Expression array datasets GSE21257 (including 53 osteosarcoma biopsy samples) [7], GSE42352 (including 84 pretreatment biopsy samples) [11], and GSE39055 (including 37 osteosarcoma biopsy samples) [8] with accompanying clinical information were obtained from the Gene Expression Omnibus [12] (GEO, http://www.ncbi.nlm.nih.gov/geo/).

### Cell lines and cell culture

All cell lines were confirmed to be mycoplasma-free based on the VenorGeM OneStep detection kit (Minerva Biolabs). The cell lines U2OS and SaOS2 were provided by Dr. K. Matsuda, Department of Computational Biology and Medical Sciences, Graduate School of Frontier Sciences, The University of Tokyo, while the HuO3N1 line was provided by the Department of Cell Biology, Okayama University Graduate School of Medicine, Dentistry, and Pharmaceutical Sciences. G292 was provided by Dr. J. Toguchida, Institute for Frontier Medical Sciences, Field of Clinical Application Department of Tissue Regeneration, Kyoto University. IMR-32 was provided by RIKEN Cell Bank. All cell lines were grown in RPMI 1640 medium (Thermo Fisher Scientific) supplemented with heat-inactivated 10% fetal bovine serum (Gibco) and 100 units/mL penicillin‒streptomycin (Gibco) at 37 °C under a humidified 5% CO_2_ atmosphere.

### Small interfering (si)RNA transfection and cell viability assays

All in vitro experiments were conducted in triplicate and repeated at least twice to validate the results to ensure the reproducibility and validity of the results. For siRNA knockdown assays, osteosarcoma cell lines (U2OS, HuO3N1, SaOS2, or G292) were seeded in 96-well plates (Corning) at 3000–4000 cells per well and transfected 24 hours later with two Silencer Select siRNAs for each target gene and Silencer Select Negative control siRNA No. 1 using Lipofectamine RNAiMAX (all Thermo Fisher Scientific) [13]. In the rescue experiment, PDGF-BB (MBL QK044-0050) or EGF (Abcam ab259398) was added to the medium the day after siRNA knockdown. The number of viable cells was estimated at selected times posttransfection using the Cell Counting Kit 8 (CCK-8) according to the manufacturer’s protocol (Dojinbo). In other experiments, osteosarcoma cells were seeded as described, cultured for 24 h, and treated with itraconazole (Cayman I0732), MMP3 inhibitor VIII (Cayman 17246 [14]), and/or furin inhibitor I (Cayman 14965 [15, 16]) as indicated. Cell viability was evaluated using CCK-8 (Dojinbo). Cell cycle analysis was performed using the Cell-Clock Assay Kit (Biocolor).

### Preparation of shRNA interference vectors

A specific shRNA targeting human *C1GALT1* (shC1GALT1) was designed and subcloned, and inserted into pENTR4-H1tetOx1, CS-RfA-ETV, and CS-RfA-ETBsd vectors (RIKEN BRC) as previously reported [17], while a nontargeting control shRNA was designed against luciferase (shLuc). The target sequences are provided in Supplemental Table S1. Cell lines were transfected as described in the previous sections.

### Production and transduction of lentivirus

For the production of lentiviral vectors, HEK293T cells were transiently transfected with the packaging construct (pCAG-HIVgp), the VSG-G and Rev-expressing construct (pCMV-VSV-G-RSV-Rev), and the self-inactivating lentiviral vector construct as previously reported [18]. The viral supernatant was then collected and concentrated using Amicon Ultra15 Centrifugal Filter Units (100 K) (Millipore, C7715). Following measurements of viral titer using the Lenti-X p24 Rapid Titer Kit (Takara Bio, 632200) and Lenti-X GoStix Plus (Takara Bio, 631280), G292 cells were infected and selected by continuous culture in blasticidin S (Cayman, 14499). For the in vitro study of shRNA effects, the cells were incubated with doxycycline for 2 days in the wells of 6-well plates (Corning) or 4 days in the wells of 96-well plates. Knockdown of *C1GALT1* was confirmed by Western blotting. The number of viable cells was estimated using CCK-8 (Dojinbo) according to the manufacturer’s instructions.

### Xenograft mouse model

To establish a xenograft mouse model of osteosarcoma, male NOG mice aged 6 to 8 weeks (CLEA Japan, Inc.) were injected in the flanks with 1 × 10^6^ G292 cells infected with either shC1GALT1 lentiviral vector (experimental group) or shLuc lentiviral vector (control group). In all experiments, experimental and control groups were formed by selectively injecting individual littermates with shC1GALT1 or shLuc. Tumors were measured with a caliper, and volume was calculated according to the formula (length × width^2^)/2. Mice were given oral doxycycline through drinking water starting on the day the tumor size reached 100 mm^3^. Ten mice were assigned to each group. Randomization and blinding were not used. Mice were treated for up to 34 days after tumor cell inoculation and euthanized if the tumor size reached 2000 mm^3^. Tumors were immediately resected for immunohistochemistry.

### Immunohistochemistry

Resected tumors were formalin-fixed and paraffin-embedded using standard techniques and then cut into 5-µm-thick sections. Sections were deparaffinized, rehydrated in gradient ethanol, heated in citrate buffer (pH 6, Genostaff #ARSC6-01) for antigen retrieval, incubated in 0.3% hydrogen peroxide in methanol for 30 min to quench endogenous peroxidase activity, and incubated with G-Block (Genostaff #GB-01) and avidin/biotin blocking kit reagent (Vector #SP-2001). Blocked sections were incubated with primary mouse anti-human C1GALT1 monoclonal antibody (Santa Cruz, sc-100745, dilution 1:100) at 4 °C overnight, washed, incubated with biotin-conjugated goat anti-mouse IgG (Vector #BA9200) for 30 min at RT, and then treated with peroxidase-conjugated streptavidin (Nichirei #426062) for 5 min. Peroxidase activity was visualized by diaminobenzidine. The sections were counterstained with Mayer’s hematoxylin, dehydrated, and then mounted under a cover glass with malinol for examination under light microscopy.

### Western blotting

Cellular proteins were extracted from cell cultures and freshly excised tumor tissue using RIPA lysis buffer, separated on 4–12% Mini-PROTEAN TGX Precast Gels (Bio-Rad), and transferred onto nitrocellulose membranes (Millipore). The membranes were then incubated with antibodies against α-tubulin (Abcam, ab7291), C1GALT1 (Santa Cruz, sc-100745), ERK (CST, 4695), phospho-Erk1/2 (CST, 4370), AKT (CST, 9272), phospho-AKT (CST, 9271), PDGFR (CST, 3169), phospho-PDGFR Tyr740 (CST, 3168), EGFR (CST, 4267), and phospho-EGFR Thr992 (CST, 2235). Membranes were washed, incubated with secondary antibodies (Cytiva, NA931 and NA934) for 1 hour, and treated with chemiluminescence reagent to visualize target protein bands.

### Comprehensive protein quantification analysis using protein arrays

The osteosarcoma cell line U2OS was seeded on 6-well plates (Corning) at 1.8–2.25 × 10^5^ per well, cultured for 24, and then transfected with siRNA as described above. Three wells were used for each condition. After 48 hours, cellular proteins were extracted using RIPA lysis buffer and used as input (200–500 μg) for the Proteome Profiler Human Phospho-Kinase Array or Human RTK phosphorylation array (RayBiotech). The chemiluminescent signal was detected using the ImageQuant LAS 4000 mini-imager (GE Healthcare). Signal blots were quantified and standardized with ImageQuant TL version 8.1 (GE Healthcare).

### Glycosylation profiling

Human glycosylation antibody arrays 493 and 507 (GAH-GCM-493 and GAH-GCM-507, RayBiotech) were used for glycosylation profiling of U2OS cells with or without prior *C1GALT1* knockdown. Briefly, U2OS cells were collected 48 hours after siRNA transfection (as detailed in the previous sections) and lysed for protein extraction. Proteins were immunolabeled on glass array slides according to the manufacturer’s protocol, and slides were scanned using GenePix4100A (Molecular Devices). After subtracting background signals and normalization to positive controls, signal intensities were compared between control and *C1GALT1* knockdown conditions. A ≥ 1.5-fold increase or ≤0.65-fold decrease in signal intensity was considered a significant difference in glycosylated protein expression provided that both signals were well above the background (mean background +2 standard deviations).

### Gene expression analyses of osteosarcoma cell lines

To examine the effects of *C1GALT1* knockdown on the gene expression profile, U2OS cells were transfected with siRNAs as described and collected 48 hours later for RNA extraction. To evaluate the effect of itraconazole treatment on gene expression, U2OS cells were treated with 2.5 μM itraconazole or vehicle (DMSO) for 48 h starting 24 h after seeding. After the indicated treatment, RNA was extracted using NucleoSpin RNA (MACHEREY-NAGEL), and libraries for RNA sequencing were prepared using the NEBNext Ultra RNA Library Prep kit from Illumina (New England Biolabs). Next-generation sequencing was performed using the Illumina HiSeq 2000 or 2500 platform with a standard 100-bp paired-end read protocol according to the manufacturer’s instructions. Reads were aligned, quality checked, and counted using our Genomon pipeline (http://genomon.hgc.jp/exome/en/index.html). Read counts were normalized by variance-stabilizing transformation using the R package DESeq2 application [19]. Differential expression was analyzed by the Wald test using negative binomial generalized linear model fitting. Gene set enrichment analysis was conducted using GSEA software version 4 [20].

### Statistical analyses

All statistical analyses were performed using R v3.5.3 software [21]. Survival times were estimated using the Kaplan–Meier method, and group values were compared using the log-rank test. Differentially expressed genes (DEGs) identified using the Wald test were used to form expression matrices. Matrices were evaluated by unsupervised consensus clustering using the ConsensusClusterPlus package (RRID:SCR_016954) to identify stable clusters [10]. Independent samples Student’s t tests were used to compare functional assay results for proliferation and tumor size. Beta regression was used for comparisons of percentages that sum to 100%. A *P* < 0.05 (two-tailed) was considered statistically significant for all tests. The center values of continuous variables are expressed as the median. Error bars in graphical representations are calculated and displayed as the standard error of the mean.

## Results

### Gene expression profiling stratifies osteosarcoma samples into two distinct prognosis groups

**Fig. 1:**
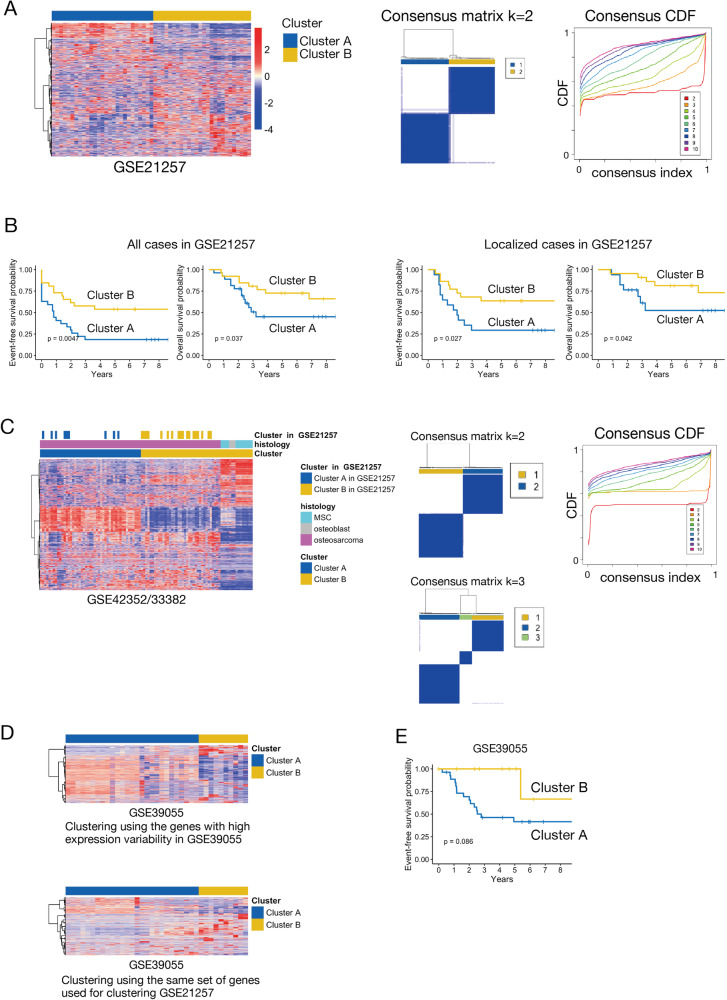
Gene expression profiling stratified human osteosarcomas into poor and fair prognosis groups. **A**, Stratification of human osteosarcomas into two clusters based on gene expression profiles. Left panel: Heatmap of gene expression levels for 51 osteosarcoma samples from the Gene Expression Omnibus (GEO) dataset GSE21257. Middle and right panels: Consensus matrix plot and CDF plot indicating that two clusters is the most suitable stratification. **B** Kaplan–Meier plots showing differences in EFS and OS rates between clusters A and B defined in Fig. 1A. The left two panels show the difference in prognosis for all cases in the GSE21257 cohort, and the right two panels show the difference in prognosis for the localized cases (cases without metastasis at the time of initial diagnosis) from clusters A and B. **C** Confirmation of clustering using an extended cohort. Left panel: Heatmap of gene expression levels for 84 samples (69 osteosarcoma samples and 15 normal bone samples) from the GSE42352/33382 cohort, which includes 27 osteosarcoma samples from the GSE21257 cohort. Consensus matrix (middle) and CDF plot (right) indicating that dividing the samples into two or three clusters is most appropriate. The clusters defined in Fig. 1A are presented in the top row. MSC; mesenchymal stem cell. **D** Heatmap of gene expression levels for 37 samples from the independent GSE39055 gene set. Genes with high expression variability defined two clusters (top). Stratifying cases using the same set of genes used for clustering GSE21257 defined two similar clusters (bottom). **E** Kaplan–Meier plot showing the difference in EFS rates for each cluster defined in Fig. 1D.

To identify novel therapeutic targets for osteosarcoma, we first analyzed the expression array dataset of 53 pretreatment biopsy samples (GSE21257 [7]) deposited in the publicly available Gene Expression Omnibus (GEO) [12]. Unsupervised consensus clustering indicated that the dataset was best divided into two stable clusters, A and B (Fig. 1A). The clinical and demographic characteristics of patient groups A and B corresponding to these clusters are summarized in Supplementary Table S2. While there were no significant differences in age, sex ratio, or histological type between patients segregated by clustering, EFS and overall survival (OS) were significantly shorter in the patient group yielding cluster A, as evidenced by Kapan–Meier analysis and log-rank test (Fig. 1B). There were also more cases with metastasis at the time of initial diagnosis in cluster A than in cluster B (10/27, 37% vs. 4/26, 15%). However, the difference did not reach statistical significance (*p* = 0.119 by Fisher’s exact test). Furthermore, prognosis was still significantly poorer (shorter EFS and OS) in cluster A patients without metastasis at diagnosis than in cluster B patients without metastasis at diagnosis, indicating that the difference in prognosis was unrelated to the initial condition (Fig. 1B) and suggesting that cluster A is indicative of a more aggressive disease subtype.

To ensure the validity of our findings, we conducted consensus clustering analyses on the GSE42352/33382 [11] dataset consisting of 84 samples in total, of which 27 samples overlapped with the GSE21257 dataset and an additional 15 normal bone samples. Consistent with the initial analyses, samples from GSE42352/33382 were divided into two stable clusters (Fig. 1C). Samples overlapping with GSE21257 clustered in cluster B (with good prognosis) in the previous analysis of GSE21257 were clustered in the same cluster as the 15 normal bone samples in the analysis of GSE42532/33382.

Consensus clustering analysis of another independent cohort, GSE39055 [8], also yielded two clusters using its top DEGs (Fig. 1D, top). To confirm the universality of the clustering, we also tried to cluster GSE39055 using the genes used to cluster GSE21257, the top DEGs in GSE21257. When clustering using this gene list, the sample was divided in the same way as when using the top DEGs of GSE39055 (Fig. 1D, bottom). While the EFS curve for one cluster trended lower than the other, suggesting a potential difference in prognosis between the two clusters, the difference did not reach statistical significance by log-rank test (Fig. 1E, *p* = 0.086). Nonetheless, these analyses indicate that more aggressive osteosarcoma cells possess a distinct gene expression profile compared to less aggressive cells.

### Extraction of genes upregulated in poor prognosis clusters

Genes upregulated in this poor prognosis cluster may contribute to tumor aggression and thus serve as prognostic biomarkers and therapeutic targets. We targeted two independent cohorts, GSE21257 and GSE39055, to identify the most promising targets. Initially, we identified genes in each cohort that were significantly upregulated within the poor prognosis cluster using the Wald test (*p* < 0.05). For each identified gene in the GSE21257 cohort, we divided all samples in GSE21257 into three expression level-based groups: high (top third), medium, and low (bottom third). By comparing the EFS rates between the high-expression and low-expression groups, we determined genes significantly associated with poor prognosis using the log-rank test (*p* < 0.05). We applied a similar analytical approach to the GSE39055 cohort, leading to the identification of another gene set. These analyses identified seven genes that consistently met all criteria across these steps (Supplementary Table S3).

### Knockdown of the *C1GALT1* gene inhibits osteosarcoma cell proliferation in vitro and in vivo

**Fig. 2:**
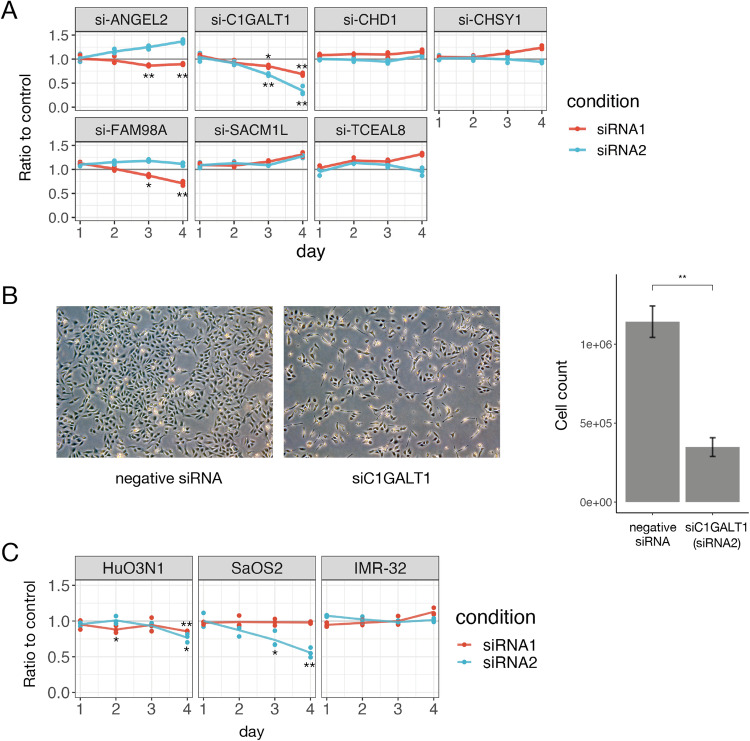
Knockdown of *C1GALT1* reduced the osteosarcoma cell proliferation rate in vitro. **A**, The results of cell viability assays for the U2OS cell line using the Cell Counting Kit 8 (CCK-8). Two targeted siRNAs with distinct sequences were used to suppress the expression of each candidate gene. The line plots show the ratio of CCK-8 fluorescence intensity for the gene knockdown group relative to the matched negative control group (transfected with negative siRNA) for each day of culture. Note the substantial reduction in the ratio, indicative of slower proliferation, induced by *C1GALT1* knockdown. Statistical significance is indicated as follows: *p < 0.05, **p < 0.01. **B** Knockdown of *C1GALT1* reduced the number of U2OS cells and changed their appearance in vitro. Left, microscopy images of U2OS cells transfected with *C1GALT1* siRNA or negative control siRNA 120 hours after transfection. Right, cell counts of U2OS cells 120 hours after the transfection of negative siRNA or siC1GALT1. **C** Knockdown of *C1GALT1* reduced the proliferation rate of another two osteosarcoma cell lines but had no effect on the proliferation of the neuroblastoma cell line IMR-32, which expressed little C1GALT1.

To elucidate the potential of these 7 genes as therapeutic targets, each was knocked down in osteosarcoma cell lines, and the effects on cell proliferation were compared to the corresponding control. First, we performed siRNA knockdown of two separate target sequences for each gene in the U2OS cell line, as this line proliferates rapidly and can be transfected with high efficiency using Lipofectamine. Of the seven genes examined, knockdown of the *C1GALT1* gene most strongly inhibited proliferation, as evidenced by the CCK-8 assay (Fig. 2A, B, and Supplementary Fig. S1A–C). Furthermore, *C1GALT1* knockdown also suppressed the proliferation of HuO3N1 and SaOS2 cell lines, both of which also reliably express *C1GALT1* according to the Cancer Cell Line Encyclopedia [22] (Fig. 2C and Supplementary Fig. S1A). In contrast, no apparent inhibition was observed following *C1GALT1* knockdown in IMR-32 neuroblastoma cells, a cell line with less intense *C1GALT1* expression (Fig. 2C and Supplementary Fig. S1A).

**Fig. 3:**
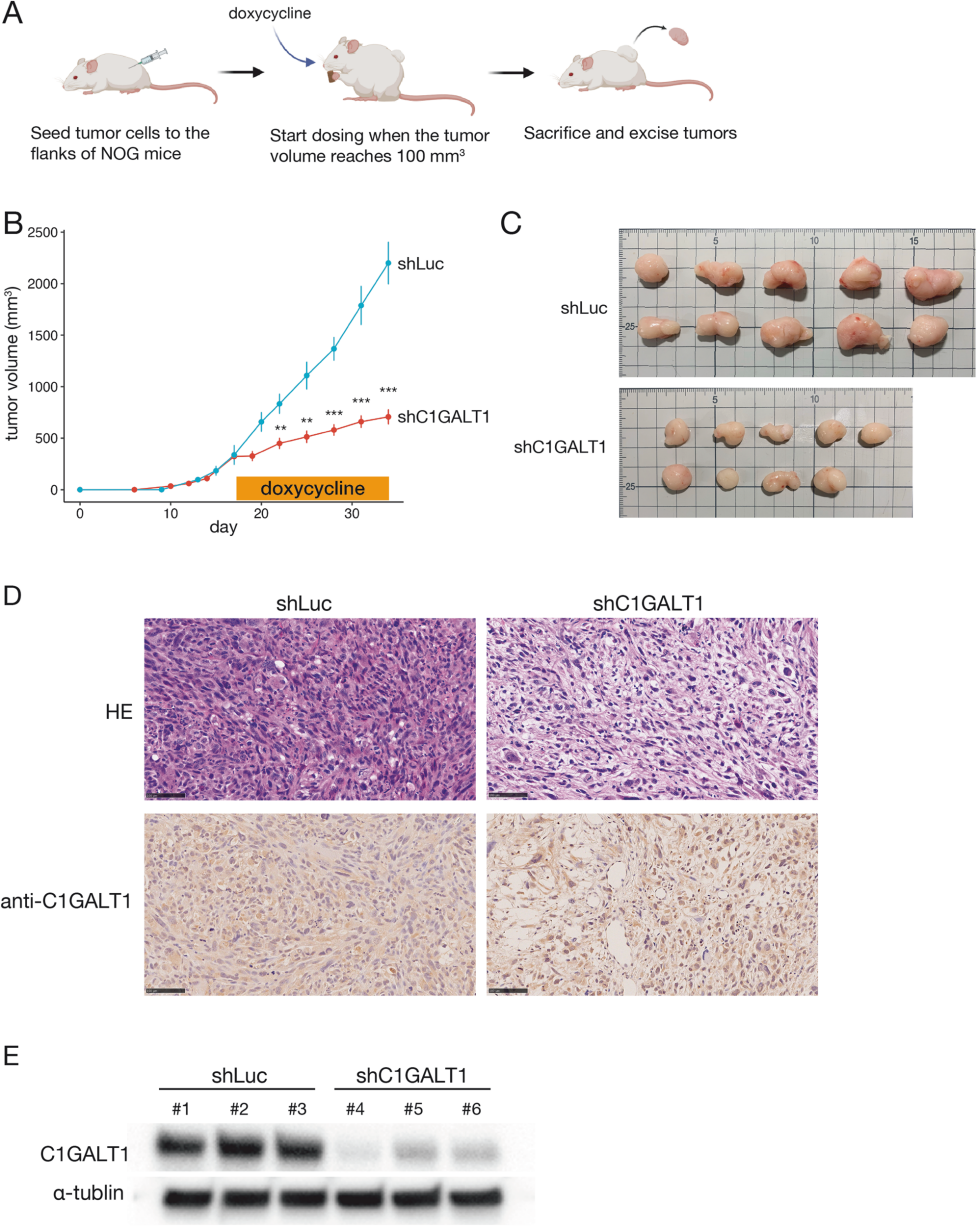
Knockdown of *C1GALT1* reduced osteosarcoma cell proliferation and tumor growth rates in mice. **A** Schema of the in vivo knockdown study (created using BioRender.com). The experiment was performed in decuplicate (ten tumors in ten mice per condition). Administration of doxycycline (Dox) to induce shRNA-mediated C1GALT1 knockdown was initiated on the day the tumor size reached 100 mm^3^. **B** Differences in tumor size between mice injected with G292 cells harboring (Tet)-inducible *C1GALT1*-targeted shRNA (shC1GALT1) or control G292 cells (transfected with shLuc). Error bars represent the standard deviations. Statistical significance is indicated as follows: **p < 0.01, ***p < 0.001. **C** Macroscopic images of excised tumors demonstrating that *C1GALT1* knockdown substantially reduced the growth rate. **D** Microscopy images of tumor sections stained with hematoxylin-eosin or anti-C1GALT1 antibody. Knockdown of *C1GALT1* markedly reduced tumor cell density, but there was no significant change in C1GALT1 immunoexpression among surviving cells. **E** A Western blot image showing the reduction in C1GALT1 protein levels in vivo. The protein extracted from tumors removed from the mice was used.

To validate this association between C1GALT1 expression and osteosarcoma cell proliferation in vivo, we selected G292 as the cell with the most stable tumor engraftment in subcutaneous injection into mice and confirmed that knockdown of *C1GALT1* by siRNA reduced the growth of G292 (Supplementary Fig. S2A). We established G292 sublines stably expressing a doxycycline-inducible short hairpin RNA (shRNA) targeting *C1GALT1* or a control construct by lentivirus transfection (Supplementary Fig. S2B–D) [18] and compared tumor growth rates following inoculation of NOD/Shi-scid, IL-2Rγ KO Jic (NOG) mice. Consistent with culture findings, the knockdown of *C1GALT1* by doxycycline treatment significantly reduced the tumor growth rate compared to mice inoculated with the control cell line (Fig. 3A–C). Furthermore, microscopic examination of excised tumors revealed a substantial decrease in cell density and deformation of tumor cells by *C1GALT1* knockdown. However, there was no obvious decrease in C1GALT1 immunostaining (Fig. 3D) even though a clear decrease in protein was observed in Western blot (Fig. 3E), suggesting that cells with knockdown of the C1GALT1 gene are reduced by cell death. These results indicate that *C1GALT1* is a major promoter of osteosarcoma cell proliferation and, thus, a promising candidate therapeutic target.

### Itraconazole reduces C1GALT1 protein expression in osteosarcoma cells and inhibits proliferation

**Fig. 4:**
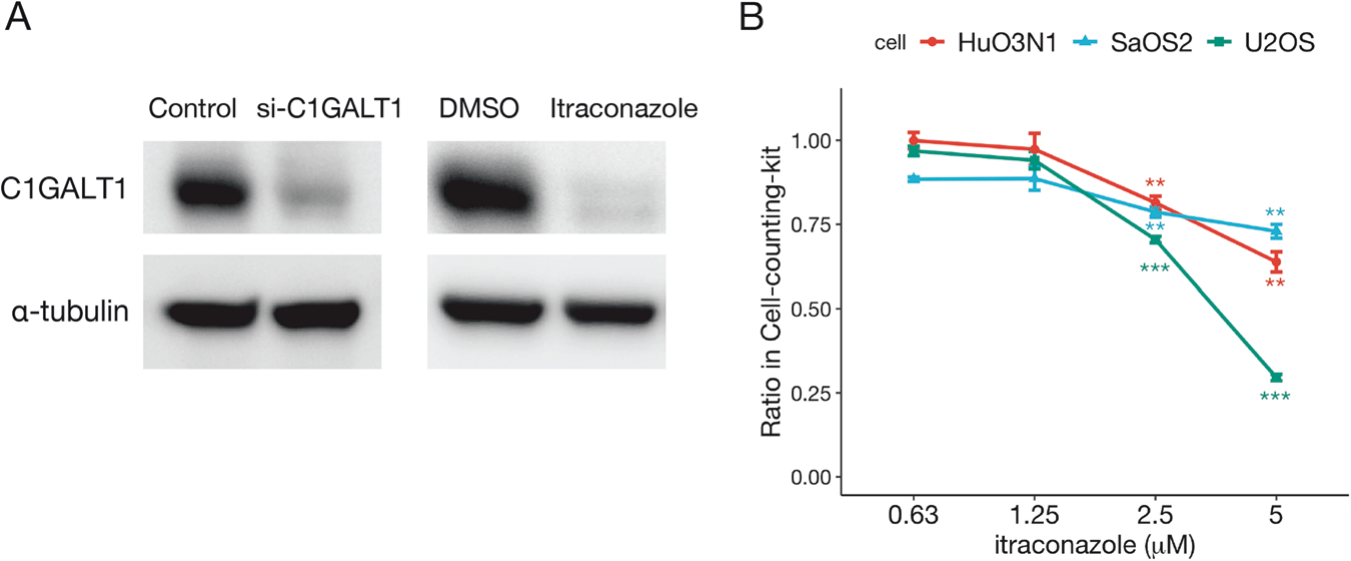
The C1GALT1 inhibitor itraconazole suppressed the proliferation rates of multiple osteosarcoma cell lines. **A**, Western blot showing that both a siRNA targeting *C1GALT1* (left) and itraconazole (2.5 μM, right) reduced C1GALT1 protein expression in U2OS cells. Alpha-tubulin was used as a gel loading control. **B** Itraconazole reduced the cell proliferation rates of 3 osteosarcoma cell lines as measured by CCK-8 assay. Statistical significance is indicated as follows: **p < 0.01, ***p < 0.001.

It has been reported that the azole antifungal drug itraconazole [23] inhibits the polymerization and function of C1GALT1 protein in some carcinomas [24], so we examined its effects on C1GALT1 expression in osteosarcoma cells. Indeed, itraconazole reduced C1GALT1 protein expression in U2OS cells (Fig. 4A). Moreover, consistent with the knockdown experiments, itraconazole dose-dependently reduced the proliferation rates of all three osteosarcoma cell lines examined (Fig. 4B).

### Expression of *C1GALT1* is required to maintain ERK pathway activation in osteosarcoma cells

**Fig. 5:**
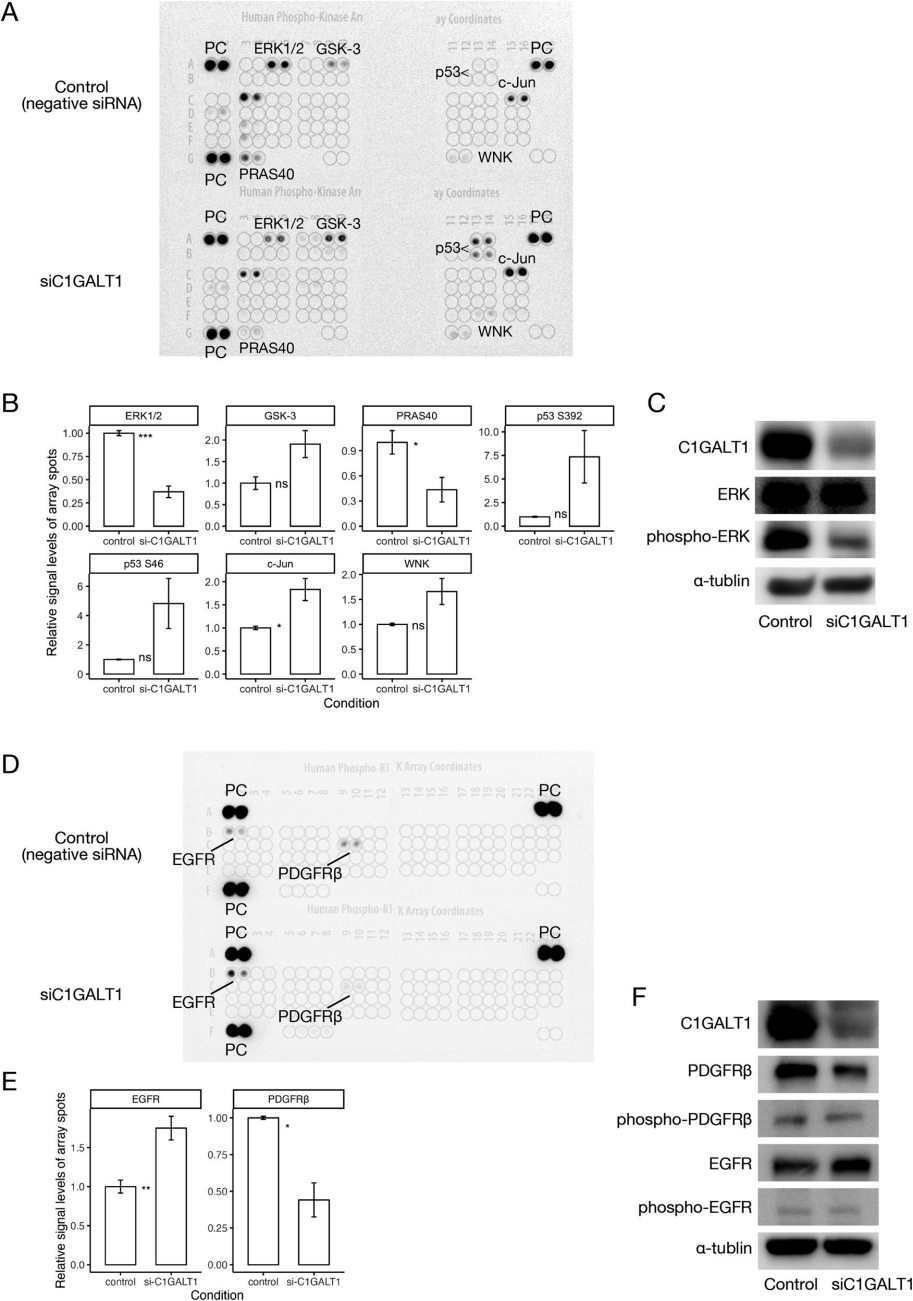
Knockdown of *C1GALT1* attenuated receptor tyrosine kinase–ERK signaling in osteosarcoma cells. **A** Changes in protein kinase phosphorylation levels induced by *C1GALT1* knockdown as detected using a Human Phospho-Kinase Array Kit. The chemiluminescent signal was detected using the ImageQuant LAS 4000 mini-imager (GE Healthcare). PC, positive control. **B** Relative signal levels of array spots in Fig. 5A. There were two blots corresponding to each protein for one membrane set, and the experiment was repeated twice for each membrane. Comparisons between conditions were determined using a t-test. Statistical significance is indicated as follows: *p < 0.05, **p < 0.01, ***p < 0.001. NC negative control, KD knockdown. **C** Western blot confirming the effects of *C1GALT1* knockdown on protein kinase expression. **D** Changes in receptor tyrosine kinase phosphorylation levels induced by *C1GALT1* knockdown as detected using the Human Phospho-RTK Array Kit. **E** Relative signal levels of array spots in Fig. 5D. **F** Western blot confirming the effects of *C1GALT1* knockdown on receptor tyrosine kinases.

The *C1GALT1* gene encodes an enzyme required for the initial step of O-glycosylation [25], a posttranslational modification required for the proper function of many proteins [26]. Furthermore, O-glycosylation has been reported to regulate various intracellular phosphorylation pathways in carcinoma [24, 27–30]. Consistent with these findings, the knockdown of *C1GALT1* in U2OS cells reduced ERK phosphorylation (p-ERK) and enhanced phosphorylation (activation) of the tumor suppressor p53, as evidenced by phosphoprotein arrays and Western blotting (Fig. 5A–C, Supplementary Table S4). Furthermore, *C1GALT1* knockdown reduced the phosphorylation of platelet-derived growth factor receptor beta (PDGFRβ), an upstream activator of ERK, as revealed using a receptor tyrosine kinase array kit (Fig. 5D–F, Supplementary Table S4). Western blotting confirmed the reduction in total PDGFRβ expression, not just its phosphorylated form (Fig. 5F). These results indicate that C1GALT1 promotes osteosarcoma cell proliferation by positively regulating the PDGFRβ–p-ERK signaling pathway. To further validate the link between the ERK phosphorylation pathway and C1GALT1, we performed a rescue experiment using PDGF-BB and EGF. Administration of PDGF-BB, but not EGF, after *C1GALT1* knockdown partially relieved the growth inhibition caused by the knockdown (Supplementary Fig. S3A). Western blotting confirmed that the decrease in phosphorylated ERK by *C1GALT1* knockdown was also relieved by PDGF-BB administration (Supplementary Fig. S3B). Besides, as another finding of the receptor tyrosine kinase array, there was an enhancement of phosphorylated EGFR by knockdown of C1GALT1 (Fig. 5D and E). Western blotting showed increased total EGFR (Fig. 5F), suggesting negative feedback associated with the attenuated phosphorylated ERK pathway. These results suggest that *C1GALT1* knockdown causes attenuation of the PDGFR-mediated ERK phosphorylation pathway, resulting in EGFR enhancement as feedback, thus indicating that C1GALT1 is required for the maintenance of the phosphorylated ERK pathway.

Glycome array analysis also revealed that *C1GALT1* knockdown slightly attenuated whole-cell glycosylation (Supplementary Fig. S4). However, the glycosylation status of individual proteins was significantly enhanced in some cases and attenuated in others (Supplementary Table S5).

### C1GALT1 is essential for cell cycle progression in osteosarcoma cells

**Fig. 6:**
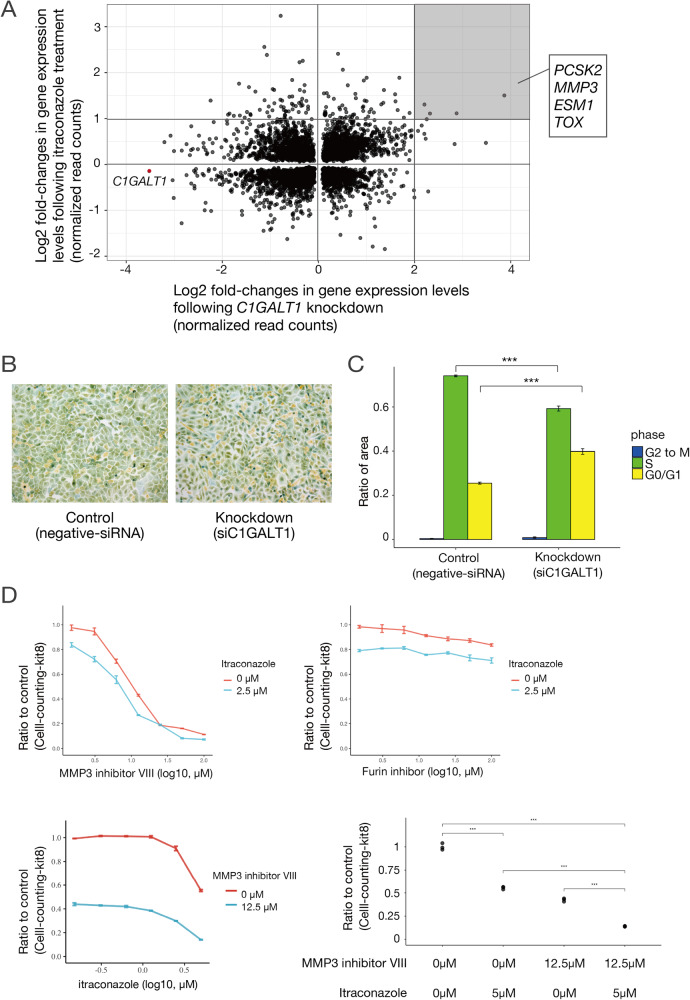
Effects of *C1GALT1* knockdown and pharmacological inhibition on the gene expression profile, cell cycle progression, and proliferation rate. **A**, Starburst plot of gene expression changes in response to *C1GALT1* knockdown and itraconazole treatment as assessed by RNA sequencing. The X-axis represents the log2 fold-change in RNA-seq read counts due to knockdown, while the Y-axis represents the log2 fold-change in RNA-seq read counts due to drug treatment. Annotations are provided for the plot representing *C1GALT1* and the top four genes with increased expression in both knockdown and drug treatment conditions. **B** Microscopy images of tumor cells stained using the Cell-Clock Assay Kit to reveal changes in cell cycle stage distribution due to *C1GALT1* knockdown compared to control cells treated with negative siRNA. **C** Bar graph of cell cycle phase distribution based on the staining patterns in Fig. 6B. Knockdown of *C1GALT1* increased the proportion of cells in G0/G1 phase. Statistical analysis was conducted using beta regression to account for the proportional nature of the data, with *** indicating *p* < 0.001. **D** Effect of MMP3 inhibitor VIII with or without itraconazole (top left), furin inhibitor with or without itraconazole (top right), and itraconazole with or without MMP3 inhibitor VIII on U2OS cell proliferation rate (bottom left). The bottom right panel summarizes the individual and combined effects of itraconazole and the MMP3 inhibitor VIII, revealing additivity in the suppression of proliferation. Cell proliferation was assessed using the Cell Counting Kit-8 after 72 hours of treatment.

To further assess the functions of C1GALT1 in osteosarcoma cells, we examined changes in the gene expression profiles of cell lines following *C1GALT1* knockdown or itraconazole treatment by RNA sequencing. The starburst plot in Fig. 6A illustrates these changes in gene expression by *C1GALT1* knockdown (X-axis) and itraconazole treatment (Y-axis), with genes plotted in the lower left downregulated by either intervention. According to gene set enrichment analysis [20], these downregulated genes included a disproportionate number related to the cell cycle, ribosome, or proteasome function (Supplementary Table S6). To confirm that *C1GALT1* expression is indeed involved in cell cycle maintenance, we examined the effect of *C1GALT1* knockdown on cell cycle phase distribution by redox dye staining. Knockdown of *C1GALT1* decreased the proportion of cells in the S phase (green) and increased the proportion in GO/G1 (yellow) (Fig. 6B, C), suggesting that *C1GALT1* downregulation causes cell cycle arrest in the G0/G1 phase.

### Matrix metalloproteinase 3 is upregulated by *C1GALT1* knockdown or inhibition and regulates osteosarcoma cell proliferation

Genes upregulated by *C1GALT1* knockdown or inhibition included *PCSK2*, *MMP3*, *ESM1*, and *TOX* (upper right corner of Fig. 6A and Supplementary Table S7). However, gene set enrichment analysis identified no significant KEGG pathways [31] (with a false discovery rate < 0.25). Thus, to test the possibility that inhibition of these genes/proteins also contribute to the suppression of osteosarcoma cell proliferation, we measured the effects of available inhibitors alone and in combination with itraconazole. Cotreatment with itraconazole and MMP3 inhibitor VIII (which is known as an inhibitor of MMP3 [14]), but not cotreatment with itraconazole and furin inhibitor I (which is known as an inhibitor of PCSK2 [15, 16]), additively suppressed U2OS cell proliferation (Fig. 6D).

## Discussion

In this study, we identified two distinct clinical subgroups of osteosarcoma patients, one with a substantially poorer prognosis than the other, based on differences in gene expression using consensus clustering analysis. In the group with a poorer prognosis, there were more cases with metastases at the time of initial diagnosis. However, this group demonstrated shorter EFS (e.g., time to recurrence and metastasis) and shorter OS even after eliminating all cases with preexisting metastases, suggesting a greater propensity to metastasize or a lower sensitivity to chemotherapy (or both). Furthermore, among the genes differentially expressed between these clinical groups, *C1GALT1* was upregulated in the poor prognosis group, and *C1GALT1* knockdown reduced the osteosarcoma cell proliferation rate in culture and tumor model mice. These findings suggest that *C1GALT1* is a promising therapeutic target for osteosarcoma.

The prognosis of osteosarcoma is traditionally based on the presence or absence of metastases at the initial diagnosis and the necrosis ratio of tumor cells at resection after chemotherapy [1, 32]. However, there is no established method for identifying cases with poor prognosis among those without metastasis at diagnosis. Genes that are particularly strongly expressed in the poor prognosis group may promote cellular processes related to cancer progression, such as proliferation and migration, and thus be effective targets for therapy using specific and potent protein inhibitors. Indeed, analyses of the associations between gene expression and clinical outcome and subsequent experiments combining gene knockdown or drug-induced inhibition with cell counting assays identified *C1GALT1* as a promising candidate prognostic biomarker and treatment target. Strong expression of *C1GALT1*, which encodes an enzyme required for the initial step of protein O-glycosylation [25], has been reported to predict poor prognosis of head and neck cancer, hepatocellular carcinoma, colon cancer, and ovarian cancer [24, 28, 29], possibly by regulating growth factor signaling. For instance, *C1GALT1* expression was reported to be required for fibroblast growth factor receptor phosphorylation through O-glycosylation in colon cancer tissue [28] and for binding of epidermal growth factor to its receptor (EGFR) through EGFR O-glycosylation in head and neck cancer [24].

The effects of C1GALT1 on signaling protein phosphorylation have been reported to vary depending on the malignancy [24, 28], so we also examined the relationships between C1GALT1 expression and protein phosphorylation in osteosarcoma using protein arrays. These studies revealed that, unlike other carcinomas, C1GALT1 is required for PDGFRβ-induced phosphorylation of the downstream effector ERK. Administration of PDGF-BB partially reversed the growth suppression induced by the knockdown of *C1GALT1*. Although this reversal effect was limited owing to the decreased expression of PDGFR protein, the data supports the hypothesis that C1GALT1 is involved in cell proliferation through the PDGFRβ–p-ERK signaling pathway. Since C1GALT1 is required for the stabilization of receptor-type phosphoproteins through glycosylation in other carcinomas [24, 28] and to maintain the total PDGFRβ protein expression level in osteosarcoma, we speculate that C1GALT1 may be required for the maintenance of PDGFRβ signaling through glycosylation in osteosarcoma.

Itraconazole has been reported to degrade and inhibit C1GALT1 protein in head and neck cancer [24], suggesting that this drug could mimic the antiproliferative effect of *C1GALT1* knockdown and thus potentially serve as a prototype therapeutic agent for osteosarcoma. While itraconazole did suppress the proliferation of osteosarcoma cell lines, relatively high doses were needed. However, compensatory changes in gene expression induced by C1GALT1 degradation may have sustained viable cell numbers. Consistent with this notion, gene expression analysis of cell lines after itraconazole treatment or *C1GALT1* knockdown identified both upregulated and downregulated genes. Gene-set analysis [20] revealed that the downregulated set was enriched in genes that regulate the cell cycle and ribosomal function, consistent with the observed effect on proliferation. In contrast, we speculated that some genes upregulated by itraconazole treatment or *C1GALT1* knockdown could act to compensate for the changes induced by these interventions or other antitumor treatments. In the latter case, concomitant inhibition of C1GALT1 and other upregulated genes, such as *MMP3*, could have additive or synergistic antitumor efficacy. Indeed, combining itraconazole and MMP3 inhibitor VII additively suppressed the proliferation of osteosarcoma cell lines. Therefore, combined C1GALT1 and MMP3 inhibition could be an even more effective candidate therapy for osteosarcoma. However, it is important to acknowledge that these results are derived from in vitro conditions. To ascertain the therapeutic validity of these treatments, further in vivo verification is necessary.

In conclusion, we demonstrate that osteosarcoma cases can be stratified into two prognostic groups based on gene expression profiling. Functional analyses of differentially expressed genes between cases with poor and better prognosis identified C1GALT1 as a promoter of osteosarcoma cell proliferation and thus as a potentially useful prognostic marker and therapeutic target. Our results provide novel strategies for predicting the prognosis of osteosarcoma at initial diagnosis and improving clinical outcomes.

### Supplementary information

Supplementary Fig. S1

Supplementary Fig. S2

Supplementary Fig. S3

Supplementary Fig. S4

Supplementary Tables

## Data Availability

RNA-seq data obtained in the current study have been deposited with links to BioProject accession number PRJDB17617 in the DDBJ BioProject database.
