# Supplementary figures and images for: Inhibition of the galactosyltransferase C1GALT1 reduces osteosarcoma cell proliferation by interfering with ERK signaling and cell cycle progression
Source: Cancer Gene Ther. 2024 Apr 15;31(7):1049–59. doi: 10.1038/s41417-024-00773-9 (PMC11257960; doi:10.1038/s41417-024-00773-9)

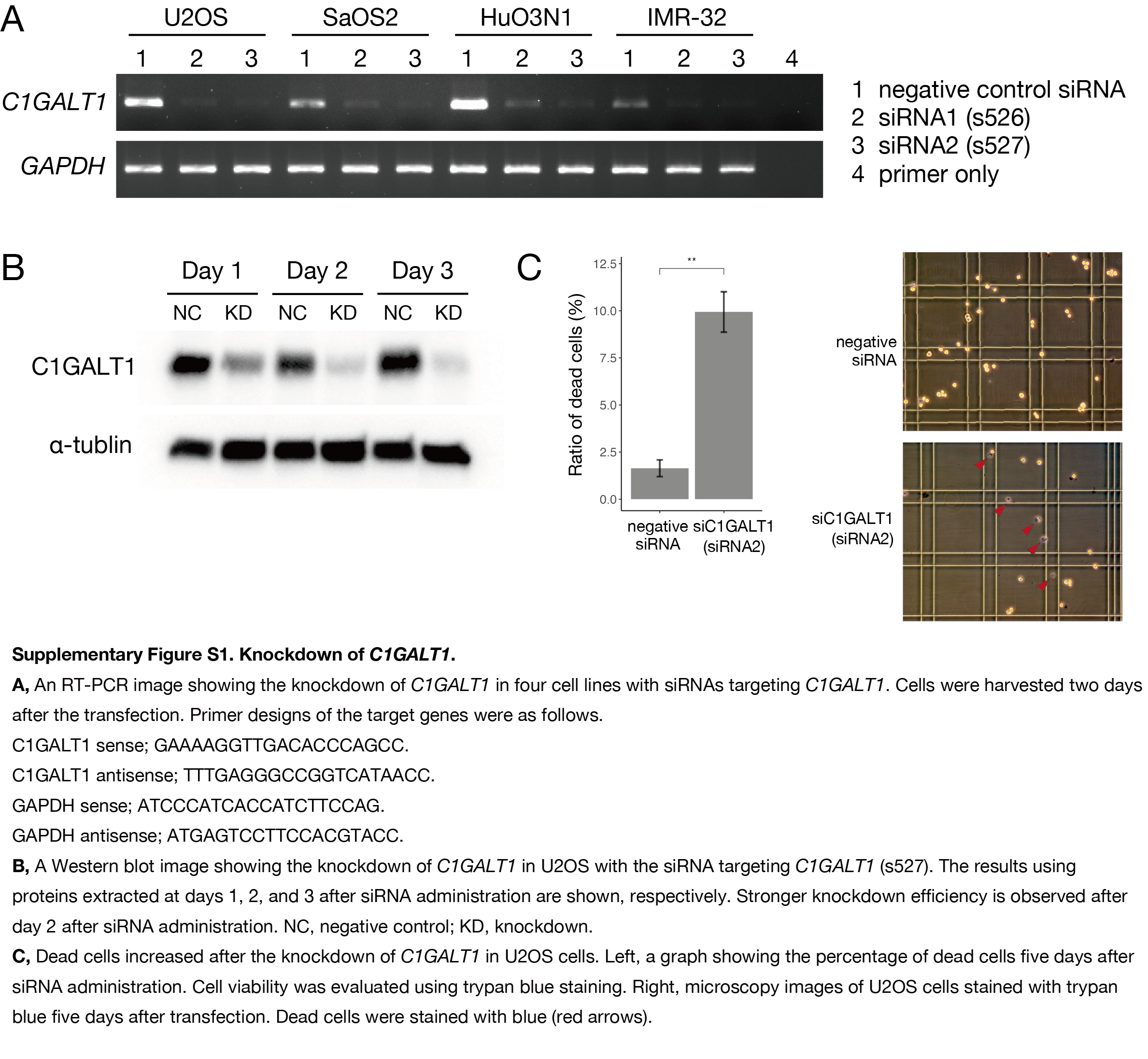

Supplement: Supplementary file 1 — Supplementary Fig. S1 [file 41417_2024_773_MOESM1_ESM.png]

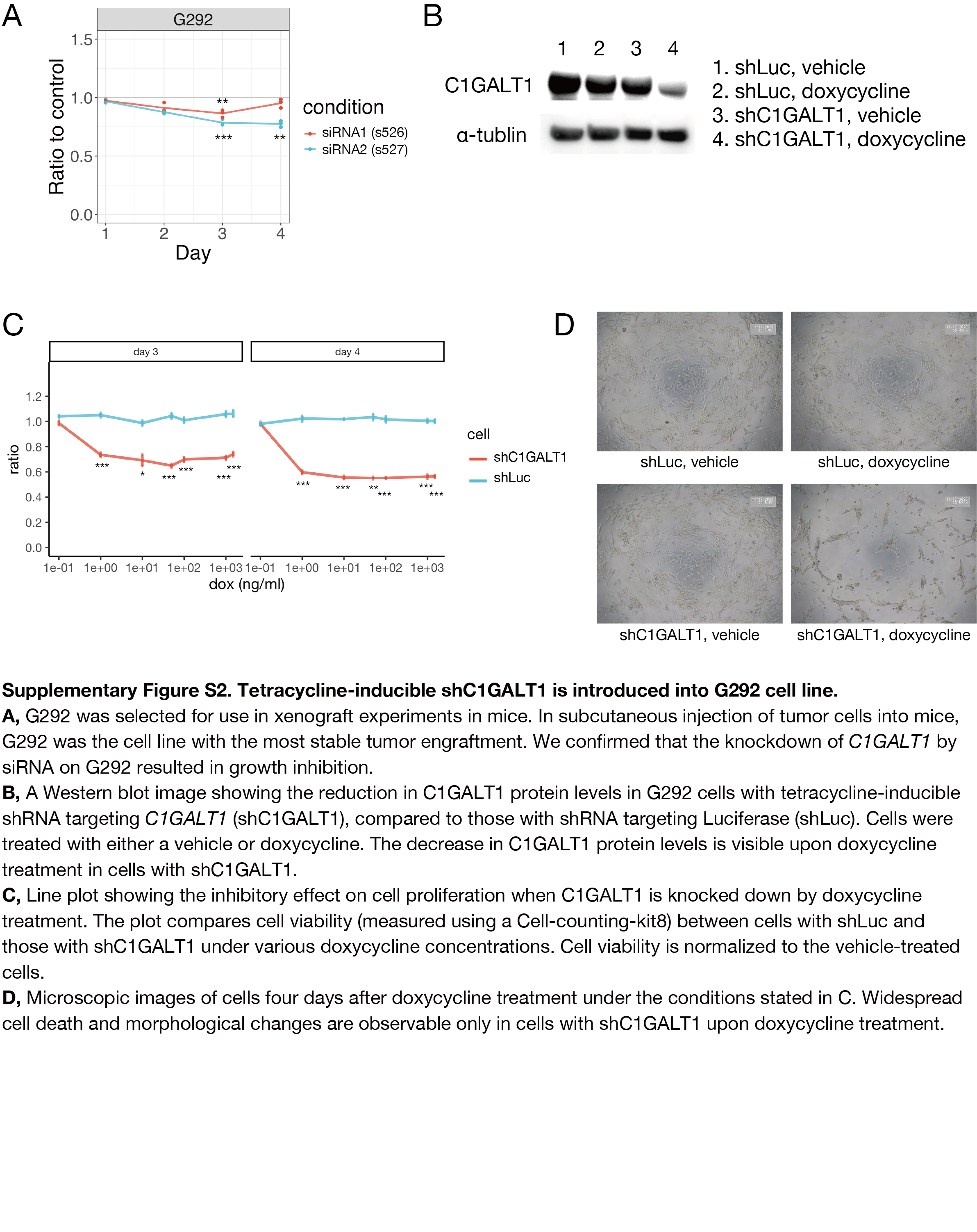

Supplement: Supplementary file 2 — Supplementary Fig. S2 [file 41417_2024_773_MOESM2_ESM.png]

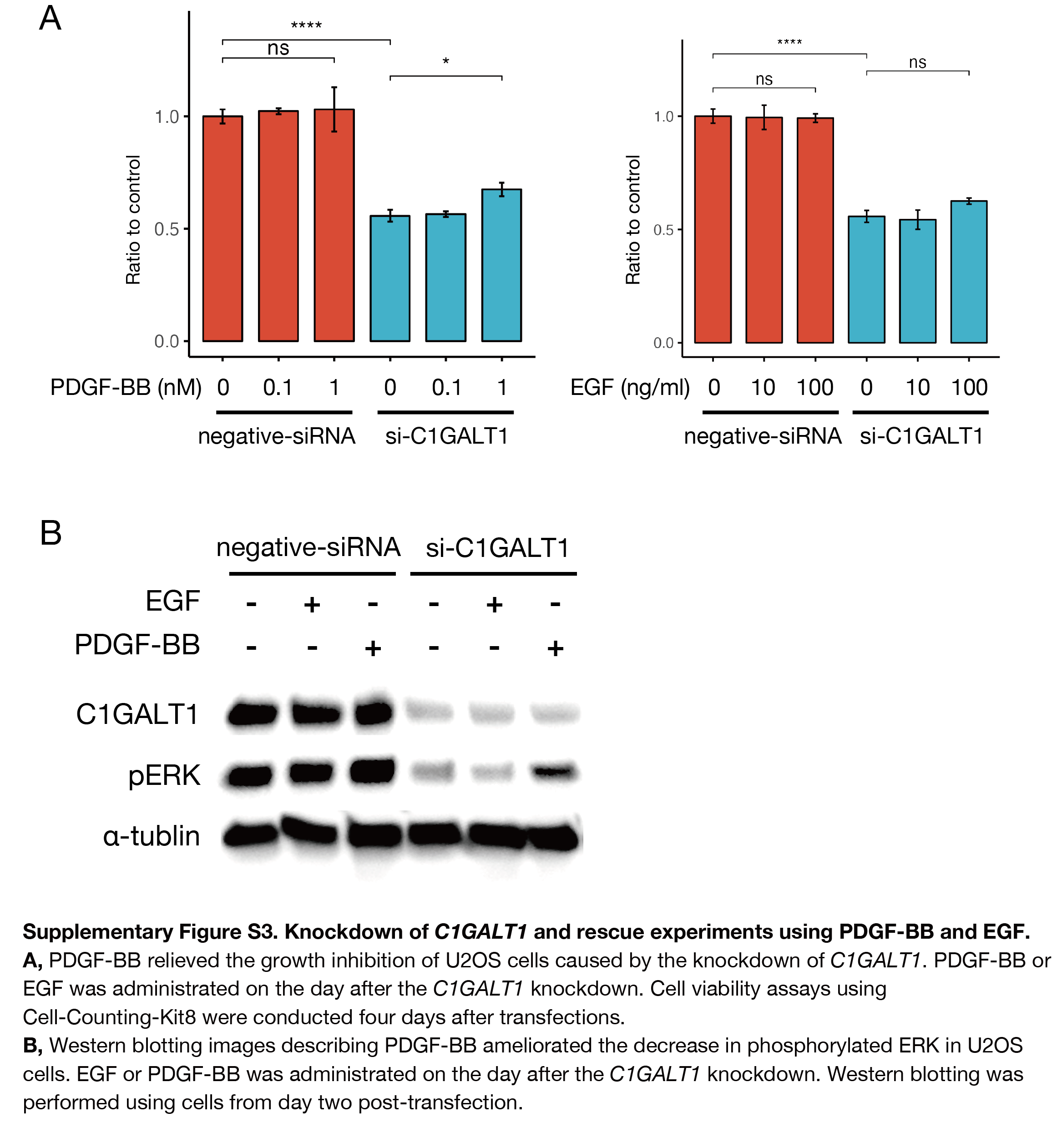

Supplement: Supplementary file 3 — Supplementary Fig. S3 [file 41417_2024_773_MOESM3_ESM.png]

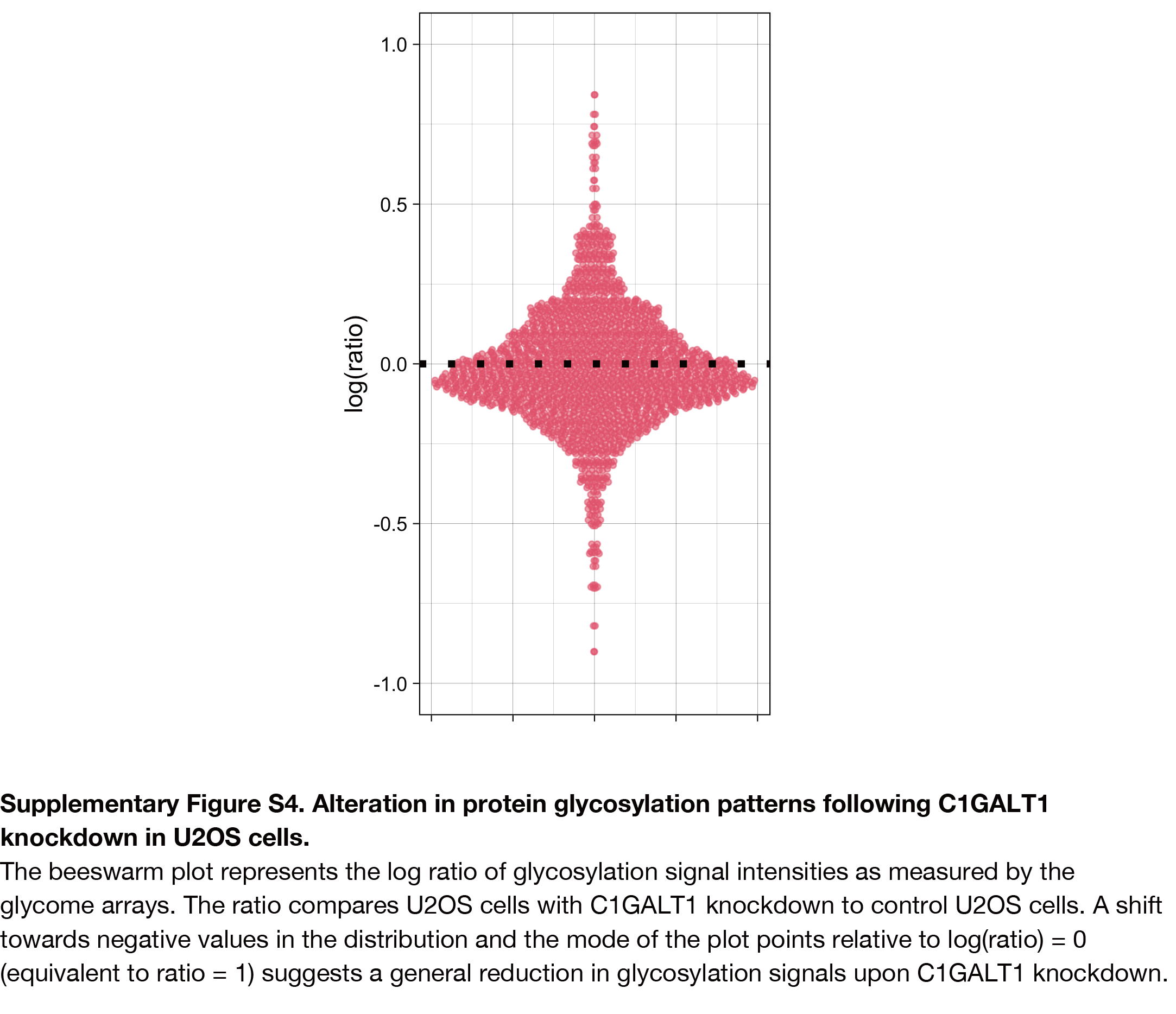

Supplement: Supplementary file 4 — Supplementary Fig. S4 [file 41417_2024_773_MOESM4_ESM.png]
